# Supplementary material for: Health Impact Assessments of Health Sector Proposals: An Audit and Narrative Synthesis
Source: Int J Environ Res Public Health. 2021 Oct 31;18(21):11466. doi: 10.3390/ijerph182111466 (PMC8582994; doi:10.3390/ijerph182111466)
Supplement: Supplementary file 1 [file ijerph-18-11466-s001.zip › ijerph-1386258-supplementary.pdf]

Supplementary File S1: Search Terms

Health Impact Assessment	Health sector
Health Impact Assessment	Health service*
	Community health*
	Tertiary health*
	Secondary health*
	Quality of health*
	Health* facility*
	Primary care*
	Primary health*
	General practice*
	Hospital*
	Health* centre*
	Health* center*
	Delivery of health*
	Nursing home*
	Residential care*
	Health planning*
	Primary health care*
	Community health service*
	Community health cent*
	Rural health service*

Supplementary File S2: Hia Websites Searched

1. HIA Connect
2. World Health Organization HIA microsite
3. The Pew Charitable Trust's Health Impact Project
4. HIA Gateway
5. Society of Practitioners of HIA
6. US Centers for Disease Control and Prevention
7. International Association for Impact Assessment

Supplementary File 3: Critical Appraisal Skills Programme (Casp 2006) Tool Analysis (Combined Reviewer Ratings)

	Health impact assessment																		
**CASP Review Questions**	**1**	**2**	**3**	**4**	**5**	**6**	**7**	**8**	**9**	**10**	**11**	**12**	**13**	**14**	**15**	**16**	**17**	**18**	**19**
**1. Was there a clear statement of the aims of the research?**																			
**2. Is the methodology appropriate?**																			
**3. Was the research design appropriate to address the aims of the research?**																			
**4. Was the recruitment strategy appropriate to the aims of the research?**																			
**5. Was the data collected in a way that addressed the research issue?**																			
**6. Has the relationship between researcher and participants been adequately considered?**																			
**7. Have ethical issues been taken into consideration?**																			
**8. Was the data analysis sufficiently rigorous?**																			
**9. Is there a clear statement of findings?**																			
**10. How valuable is the research?**	N/A	N/A	N/A	N/A	N/A	N/A	N/A	N/A	N/A	N/A	N/A	N/A	N/A	N/A	N/A	N/A	N/A	N/A	N/A

Both reviewers answered “yes”
Both reviewers answered “can’t tell”
One reviewer answered “yes”, one reviewer answered “can’t tell”
One reviewer answered “yes”, one reviewer answered “no”
